# Supplementary material for: Bisphenol A derivatives act as novel coactivator-binding inhibitors for estrogen receptor β
Source: J Biol Chem. 2021 Sep 6;297(5):101173. doi: 10.1016/j.jbc.2021.101173 (PMC8551653; doi:10.1016/j.jbc.2021.101173)
Supplement: Figures S1–S5 and Tables S1–S7 [file mmc1.pdf]

Supplementary Materials for

Bisphenol A derivatives act as novel coactivator binding inhibitors for estrogen receptor β

Masaki Iwamoto†, Takahiro Masuya†, Mari Hosose, Koki Tagawa, Tomoka Ishibashi, Keitaro Suyama, Takeru Nose, Eiji Yoshihara, Michael Downes, Ronald M. Evans, and Ayami Matsushima*

*Corresponding author. Email: ayami@chem.kyushu-univ.jp

†These authors contributed equally to this work.

This PDF file includes:

Figs. S1 to S5
Tables S1 to S7

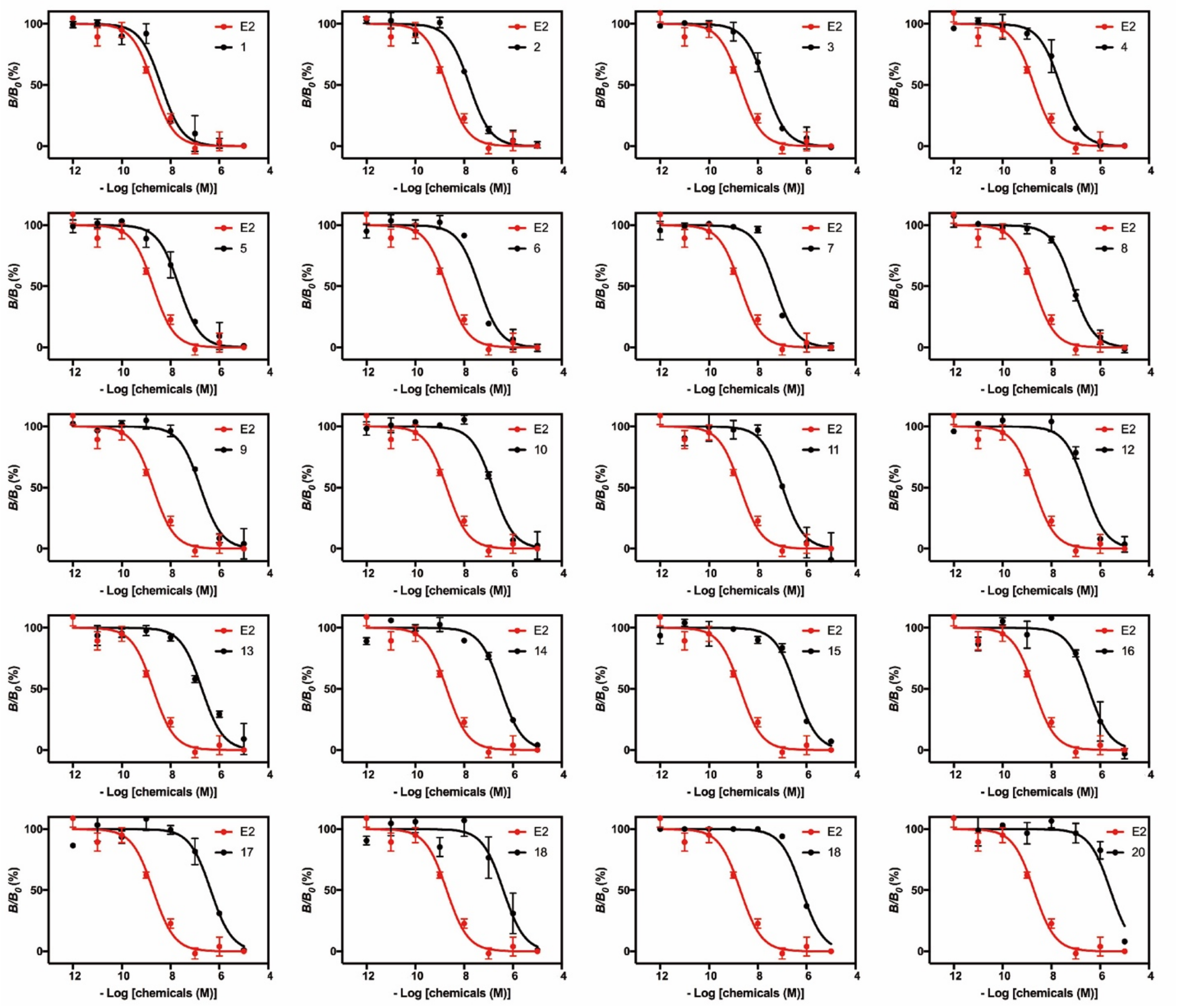

Fig. S1.

Binding curves indicated the binding ability of each bisphenol derivatives using competitive binding assays with[^{3}H]E2. *B/B_0* is the ratio of displacement by the chemical tested (*B*) against the maximum specific binding (B_0 = 100%) of [^{3}H]E2.

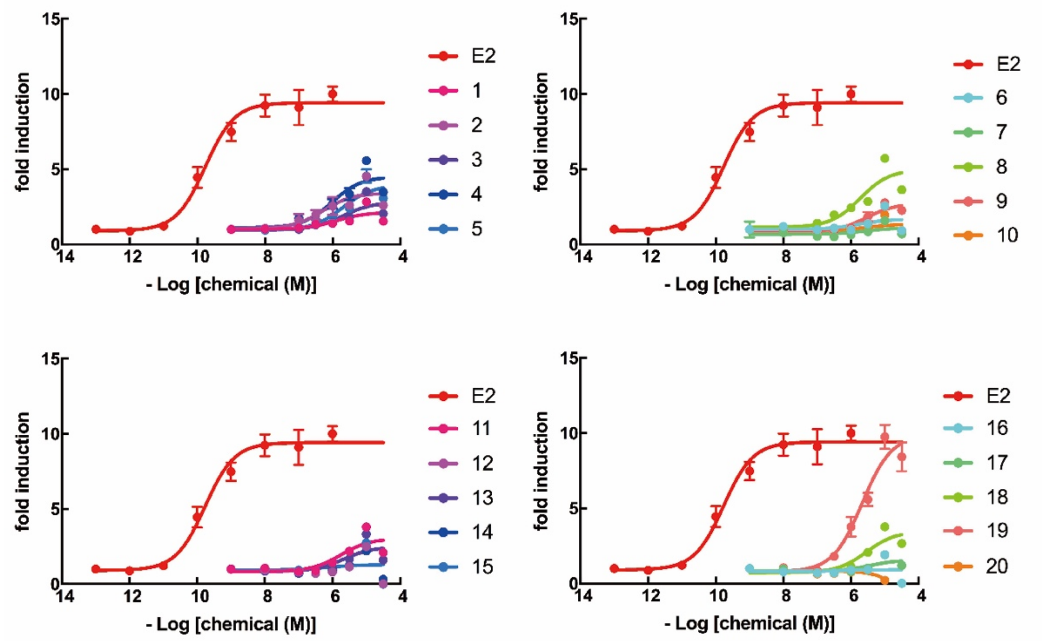

Fig. S2.

Partial agonistic activity of BPA derivatives against ERβ. BPA (chemical No.19) elicited full agonist activity, corresponding the activity induced by 1 μM E2; however, other BPA derivatives showed partial or almost no agonistic activity.

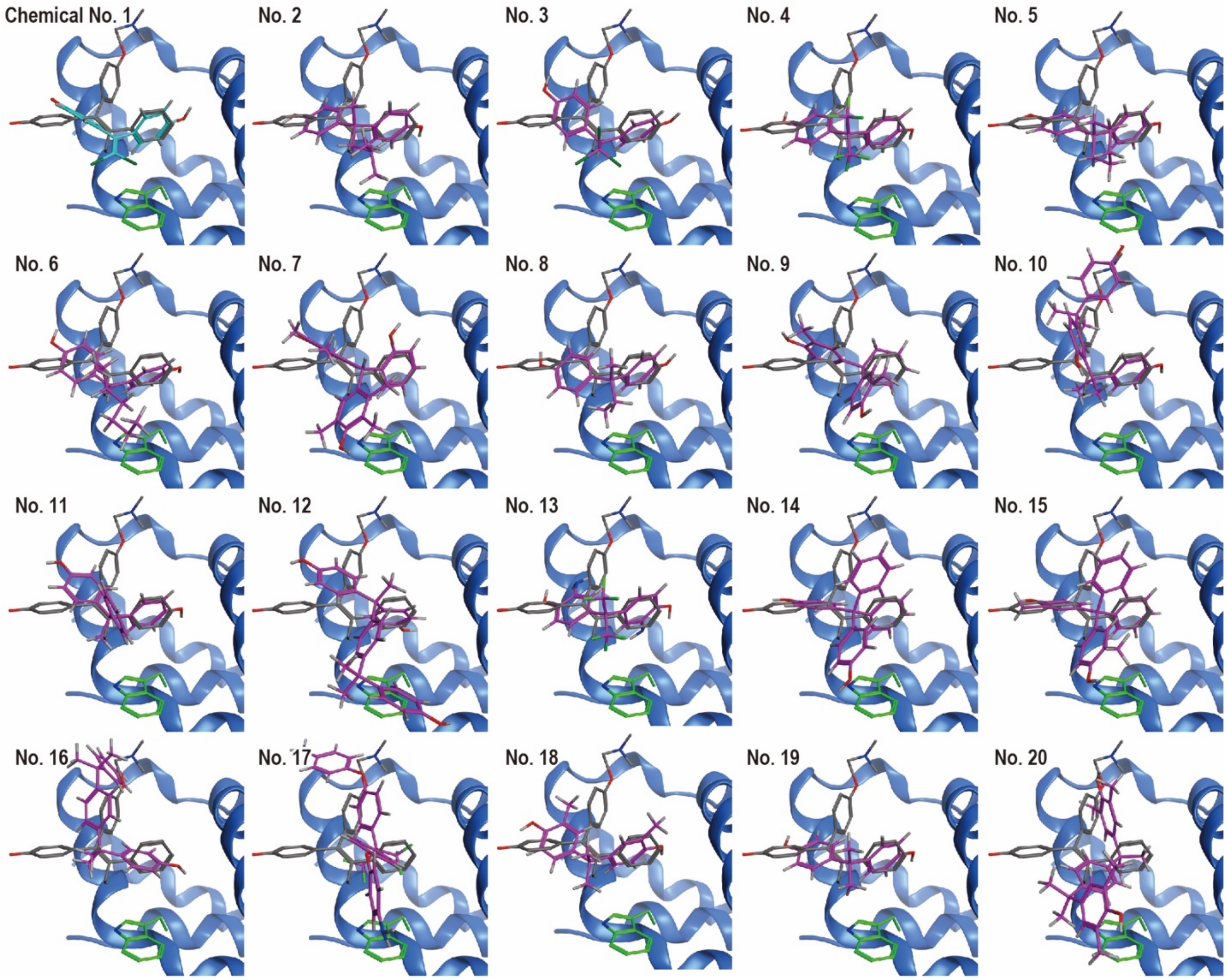

Fig. S3.

Docking simulation indicated the binding possibilities of BPA derivatives on the second binding site located on the coactivator-binding surface of ERβ. Calculated coordinates of BPC (blue stick model, No. 1) and the other BPA derivatives (magenta stick model, No. 2 to 20) were located close to W335. 4OHT in the crystal structure used for the docking simulation is indicated via a gray stick model.

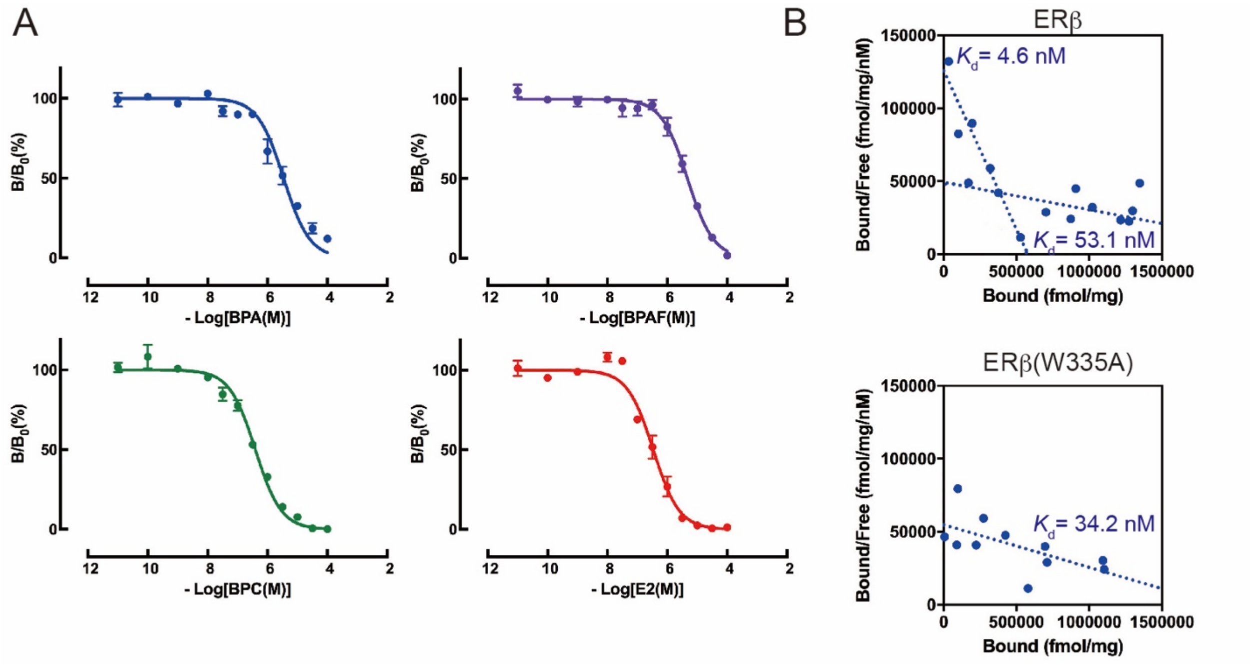

Fig. S4. Binding experiments showed that ERβ has two 4OHT binding sites, and only a single binding site for E2. (A) Competitive binding assay of ERβ(W335A) using [^{3}H]E2 showed that ERβ(W335A) retained its binding activity for E2 and other BPA derivatives. (B) Saturation binding assays using [^{3}H]4OHT estimated that ERβ has both a high and low binding sites for 4OHT, while ERβ(W335A) has only one binding site.

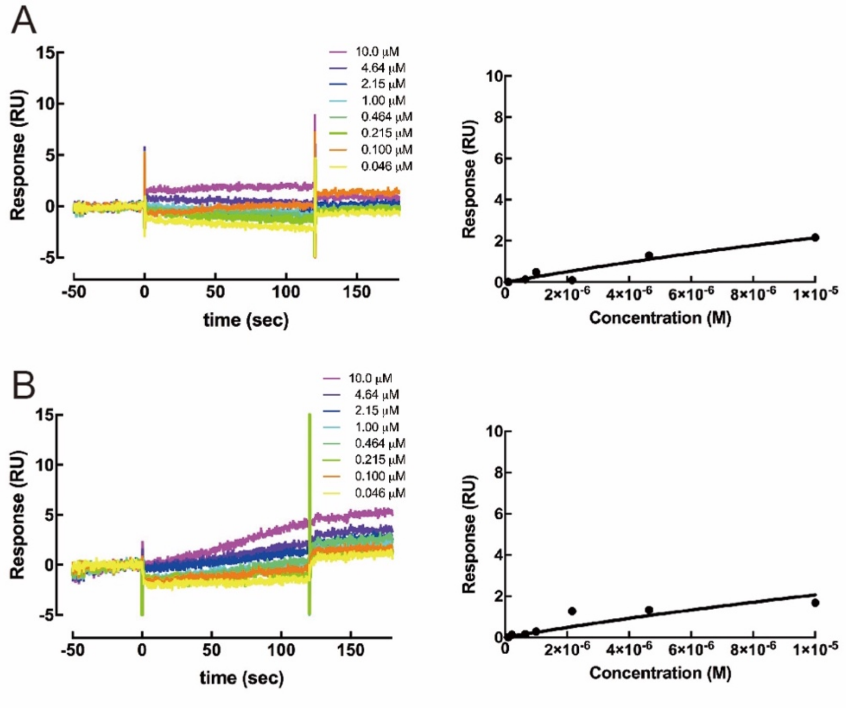

Fig. S5. A SRC1 peptide could not bind to ERβ(W335A)-LBD even with E2. (A) The binding ability of the SRC1 peptide and ERβ(W335A)-LBD was measured by surface plasmon resonance experiments using Biacore T100 with 10 μM E2 in the running buffer. (B) The binding ability of the SRC1 peptide and ERβ(W335A)-LBD was measured with 10 μM BPC.

Table S1.

CAS RNs, common names, and IUPAC names of all the chemicals whose binding ability to ERβ was analyzed using competitive binding assays in this study.

CAS RN®	common name	IUPAC name
603-44-1	4,4',4''-trihydroxytriphenylmethane	4,4',4''-methanetriyltriphenol
79-95-8	tetrachloro bisphenol A	4,4'-(propane-2,2-diyl)bis(2,6-dichlorophenol)
79-94-7	tetrabromo bisphenol A	4,4'-(propane-2,2-diyl)bis(2,6-dibromophenol)
77-40-7	bisphenol B	4,4'-(butane-2,2-diyl)diphenol
1571-75-1	bisphenol AP	4,4'-(1-phenylethane-1,1-diyl)diphenol
5613-46-7	tetramethyl bisphenol A	4,4'-(propane-2,2-diyl)bis(2,6-dimethylphenol)
79-97-0	2,2-bis(4-hydroxy-3-methylphenyl)propane	4,4'-(propane-2,2-diyl)bis(2-methylphenol)
27955-94-8	1,1',1''-tris(4-hydroxyphenyl)ethane	4,4',4''-(ethane-1,1,1-triyl)triphenol
599-64-4	4-α-cumyl phenol	4-(2-phenylpropan-2-yl)phenol
2167-51-3	bisphenol P	4,4'-(1,4-phenylenebis(propane-2,2-diyl))diphenol
14868-03-2	bisphenol C	4,4'-(2,2-dichloroethene-1,1-diyl)diphenol
80-05-7	bisphenol A	4,4'-(propane-2,2-diyl)diphenol
70-30-4	hexachlorophene	6,6'-methylenebis(2,4,5-trichlorophenol)
110726-28-8	α,α,α' -tris(4-hydroxyphenyl)-1-ethyl-4-isopropylbenzene	4,4'-(1-(4-(2-(4-hydroxyphenyl)propan-2-yl)phenyl)ethane-1,1-diyl)diphenol
2716-10-1	α,α'-bis(4-aminophenyl)-1,4-diisopropylbenzene	4,4'-(1,4-phenylenebis(propane-2,2-diyl))dianiline
57100-74-0	2,2-bis(3-cyclohexyl-4-hydroxyphenyl)propane	4,4'-(propane-2,2-diyl)bis(2-cyclohexylphenol)
24038-68-4	2,2-bis(2-hydroxy-5-biphenylyl)propane	5,5''-(propane-2,2-diyl)bis(([1,1'-biphenyl]-2-ol))
1675-54-3	2,2-bis(4-glycidyloxyphenyl)propane	2,2'-(((propane-2,2-diylbis(4,1-phenylene))bis(oxy))bis(methylene))bis(oxirane)
10192-62-8	bisphenol A diacetate	propane-2,2-diylbis(4,1-phenylene) diacetate
4162-45-2	tetrabromobisphenol A bis(2-hydroxyethyl)ether	2,2'-((propane-2,2-diylbis(2,6-dibromo-4,1-phenylene))bis(oxy))diethanol
127-54-8	2,2-bis(4-hydroxy-3-isopropylphenyl)propane	4,4'-(propane-2,2-diyl)bis(2-isopropylphenol)
3539-42-2	4,4'-isopropylidenediphenoxyacetic acid	2,2'-((propane-2,2-diylbis(4,1-phenylene))bis(oxy))diacetic acid
13080-86-9	2,2-bis[4-(4-aminophenoxy)-phenyl]propane	4,4'-((propane-2,2-diylbis(4,1-phenylene))bis(oxy))dianiline
36395-57-0	α,α'-bis(4-hydroxy-3,5-dimethylphenyl)-1,4-diisopropylbenzene	4,4'-(1,4-phenylenebis(propane-2,2-diyl))bis(2,6-dimethylphenol)
2024-88-6	2,2-bis(4-chloroformyloxyphenyl)propane	propane-2,2-diylbis(4,1-phenylene) dicarbonochloridate
32113-46-5	2,2-bis(3-sec-butyl-4-hydroxyphenyl)propane	2-butan-2-yl-4-[2-(3-butan-2-yl-4-hydroxyphenyl)propan-2-yl]phenol
620-92-8	bisphenol F	4,4'-methylenediphenol
84-16-2	hexestrol	4,4'-(hexane-3,4-diyl)diphenol
1156-51-0	2,2-bis(4-cyanatophenyl)propane	4,4'-(propane-2,2-diyl)bis(cyanatobenzene)
479-13-0	coumestrol	3,9-dihydroxy-6H-benzofuro[3,2-c]chromen-6-one
1415-73-2	barbaloin	(10*S*)-1,8-dihydroxy-3-(hydroxymethyl)-10-[(2*S*,3*R*,4*R*,5*S*,6*R*)-3,4,5-trihydroxy-6-(hydroxymethyl)oxan-2-yl]-10*H*-anthracen-9-one
961-29-5	isoliquirtigenin	(*E*)-1-(2,4-dihydroxyphenyl)-3-(4-hydroxyphenyl)prop-2-en-1-one
2467-25-6	4,4'-methylenebis(2-methylphenol)	4,4'-methylenebis(2-methylphenol)
17345-66-3	2,3,4-trihydroxydiphenylmethane	4-benzylbenzene-1,2,3-triol
75804-28-3	2,3-dimethyl-2,3-butanediamine	2,3-dimethylbutane-2,3-diamine
2081-08-5	bisphenol E	4,4'-(ethane-1,1-diyl)diphenol

2971-36-0	HPTE	4,4'-(2,2,2-trichloroethane-1,1-diyl)diphenol
83558-87-6	2,2-bis(3-amino-4-hydroxyphenyl)hexafluoropropane	4,4'-(perfluoropropane-2,2-diyl)bis(2-aminophenol)
47250-53-3	2,2-bis(3-aminophenyl)hexafluoropropane	3,3'-(perfluoropropane-2,2-diyl)dianiline
116325-74-7	2,2-bis(3-amino-4-methylphenyl)hexafluoropropane	5,5'-(perfluoropropane-2,2-diyl)bis(2-methylaniline)
1095-78-9	2,2-bis(4-aminophenyl)hexafluoropropane	4,4'-(perfluoropropane-2,2-diyl)dianiline
69563-88-8	2,2-bis[4-(4-aminophenoxy)phenyl]hexafluoropropane	4,4'-(((perfluoropropane-2,2-diyl)bis(4,1-phenylene))bis(oxy))dianiline
1478-61-1	bisphenol AF	4,4'-(perfluoropropane-2,2-diyl)diphenol
1107-00-2	4,4'-(hexafluoroisopropylidene)diphthalic anhydride	5,5'-(perfluoropropane-2,2-diyl)bis(isobenzofuran-1,3-dione)
1171-47-7	2,2-bis(4-carboxyphenyl)hexafluoropropane	4,4'-(perfluoropropane-2,2-diyl)dibenzoic acid
10224-18-7	2,2-bis(4-isocyanatophenyl)hexafluoropropane	4,4'-(perfluoropropane-2,2-diyl)bis(isocyanatobenzene)
83558-76-3	hexafluoro-2,2-diphenylpropane	(perfluoropropane-2,2-diyl)dibenzene
4221-68-5	1,1-bis(3-cyclohexyl-4-hydroxyphenyl)cyclohexane	4,4'-(cyclohexane-1,1-diyl)bis(2-cyclohexylphenol)
15499-84-0	9,9-bis(4-aminophenyl)fluorene	4,4'-(9*H*-fluorene-9,9-diyl)dianiline
184355-68-8	4,4'-(2-hydroxybenzylidene)-bis(2,3,6-trimethylphenol)	4,4'-((2-hydroxyphenyl)methylene)bis(2,3,6-trimethylphenol)
6807-17-6	4,4'-(1,3-dimethylbutylidene)diphenol	4,4'-(4-methylpentane-2,2-diyl)diphenol
3236-71-3	9,9-bis(4-hydroxyphenyl)fluorene	4,4'-(9*H*-fluorene-9,9-diyl)diphenol
88938-12-9	9,9-bis(4-hydroxy-3-methylphenyl)fluorene	4,4'-(9*H*-fluorene-9,9-diyl)bis(2-methylphenol)
74462-02-5	4,4'-(2-ethylhexylidene)diphenol	4,4'-(2-ethylhexane-1,1-diyl)diphenol
117344-32-8	9,9-bis[4-(2-hydroxyethoxy)phenyl]fluorene	2,2'-(((9H-fluorene-9,9-diyl)bis(4,1-phenylene))bis(oxy))diethanol
2362-14-3	1,1-bis(4-hydroxy-3-methylphenyl)cyclohexane	4,4'-(cyclohexane-1,1-diyl)bis(2-methylphenol)
3282-99-3	1,1-bis(4-aminophenyl)cyclohexane	4,4'-(cyclohexane-1,1-diyl)dianiline
843-55-0	bisphenol Z	4,4'-(cyclohexane-1,1-diyl)diphenol
13595-25-0	1,3-bis[2-(4-hydroxyphenyl)-2-propyl]benzene	4,4'-(1,3-phenylenebis(propane-2,2-diyl))diphenol
20601-38-1	4,4'-bicyclohexanol	[1,1'-bi(cyclohexane)]-4,4'-diol
1980-4-69	2,2-bis(4-hydroxycyclohexyl)propane	4,4'-(propane-2,2-diyl)dicyclohexanol
2433-14-6	4-cyclohexylcyclohexanol	[1,1'-bi(cyclohexan)]-4-ol
119-42-6	2-cyclohexylphenol	2-cyclohexylphenol
947-42-2	diphenylsilanediol	diphenylsilanediol
20714-70-9	4-(phenylazo)phenol	(*E*)-4-(phenyldiazenyl)phenol
501-36-0	resveratrol	(*E*)-5-(4-hydroxystyryl)benzene-1,3-diol
3127-14-8	spirobicromane	4,4,4',4'-tetramethyl-2,2'-spirobi[chroman]-7,7'-diol
2246-46-0	4-(2-Thiazolylazo)resorcinol	(*E*)-4-(thiazol-2-yldiazenyl)benzene-1,3-diol
32737-35-2	6,6',7,7'-tetrahydroxy-4,4,4',4'-tetramethyl-2,2'-spirobichroman	4,4,4',4'-tetramethyl-2,2'-spirobi[chroman]-6,6',7,7'-tetraol
269409-97-4	4-(4,4,5,5-Tetramethyl-1,3,2-dioxaborolan-2-yl)phenol	2-(4,4,5,5-tetramethyl-1,3,2-dioxaborolan-2-yl)phenol
611-99-4	4,4'-dihydroxybenzophenone	bis(4-hydroxyphenyl)methanone
90-96-0	4,4'-dimethoxybenzophenone	bis(4-methoxyphenyl)methanone
61445-50-9	2,3',4,4'-tetrahydroxybenzophenone	(2,4-dihydroxyphenyl)(3,4-dihydroxyphenyl)methanone
131-55-5	2,2',4,4'-tetrahydroxybenzophenone	bis(2,4-dihydroxyphenyl)methanone
345-92-6	4,4'-difluorobenzophenone	bis(4-fluorophenyl)methanone
131-54-4	2,2'-dihydroxy-4,4'-dimethoxybenzophenone	bis(2-hydroxy-4-methoxyphenyl)methanone
90-98-2	4,4'-dichlorobenzophenone	bis(4-chlorophenyl)methanone
2421-28-5	3,3',4,4'-benzophenonetetracarboxylic dianhydride	5,5'-carbonylbis(isobenzofuran-1,3-dione)
85-58-5	benzophenone-2,4'-dicarboxylic acid monohydrate	2-(4-carboxybenzoyl)benzoic acid

342-25-6	2,4'-difluorobenzophenone	(2-fluorophenyl)(4-fluorophenyl)methanone
611-98-3	4,4'-diaminobenzophenone	bis(4-aminophenyl)methanone
83846-85-9	4-benzoyl 4'-methyldiphenyl sulfide	phenyl(4-(p-tolylthio)phenyl)methanone
964-68-1	benzophenone-4,4'-dicarboxylic acid	4,4'-carbonyldibenzoic acid
131-53-3	2,2'-dihydroxy-4-methoxybenzophenone	(2-hydroxy-4-methoxyphenyl)(2-hydroxyphenyl)methanone
85-29-0	2,4'-dichlorobenzophenone	(2-chlorophenyl)(4-chlorophenyl)methanone
1470-79-7	2,4,4'-trihydroxybenzophenone	(2,4-dihydroxyphenyl)(4-hydroxyphenyl)methanone
835-11-0	2,2'-dihydroxybenzophenone	bis(2-hydroxyphenyl)methanone
21222-05-9	3,3'-dinitrobenzophenone	bis(3-nitrophenyl)methanone
611-79-0	3,3'-diaminobenzophenone	bis(3-aminophenyl)methanone
2958-36-3	2-amino-2',5-dichlorobenzophenone	(2-amino-5-chlorophenyl)(2-chlorophenyl)methanone
33077-87-1	2,2',4-trimethoxybenzophenone	(2,4-dimethoxyphenyl)(2-methoxyphenyl)methanone
3708-39-2	4,4'-bis(methylamino)benzophenone	bis(4-(methylamino)phenyl)methanone
119-61-9	benzophenone	benzophenone
31127-54-5	2,3,4,4'-tetrahydroxybenzophenone	(4-hydroxyphenyl)(2,3,4-trihydroxyphenyl)methanone
118-82-1	4,4'-methylenebis(2,6-di-tert-butylphenol)	4,4'-methylenebis(2,6-di-tert-butylphenol)
122-25-8	methylenedisalicylic acid	5,5'-methylenebis(2-hydroxybenzoic acid)
105391-33-1	bis(3-ethyl-5-methyl-4-maleimidophenyl)methane	1,1'-(methylenebis(2-ethyl-6-methyl-4,1-phenylene))bis(1H-pyrrole-2,5-dione)
97-23-4	2,2'-methylenebis(4-chlorophenol)	2,2'-methylenebis(4-chlorophenol)
13676-54-5	4,4'-bismaleimidodiphenylmethane	1,1'-(methylenebis(4,1-phenylene))bis(1H-pyrrole-2,5-dione)
19900-72-2	4,4'-methylenebis(2-ethyl-6-methylaniline)	4,4'-methylenebis(2-ethyl-6-methylaniline)
838-88-0	4,4'-diamino-3,3'-dimethyldiphenylmethane	4,4'-methylenebis(2-methylaniline)
101-77-9	4,4'-diaminodiphenylmethane	4,4'-methylenedianiline
101-61-1	Bis[4-dimethylamino)-phenyl]methane	4,4'-methylenebis(N,N-dimethylaniline)
88-24-4	2,2'-methylenebis(6-tert-butyl-4-ethylphenol)	6,6'-methylenebis(2-(tert-butyl)-4-ethylphenol)
101-14-4	4,4'-methylenebis(2-chloroaniline)	4,4'-methylenebis(2-chloroaniline)
5384-21-4	4,4'-methylenebis(2,6-dimethylphenol)	4,4'-methylenebis(2,6-dimethylphenol)
19430-83-2	3,4'-diaminodiphenylmethane	3-(4-aminobenzyl)aniline
119-47-1	2,2'-methylenebis(6-tert-butyl-p-cresol)	6,6'-methylenebis(2-(tert-butyl)-4-methylphenol)
1817-74-9	4,4'-dinitrodiphenylmethane	bis(4-nitrophenyl)methane
42240-73-3	Bis(4-amino-2,3-dichlorophenyl)methane	4,4'-methylenebis(2,3-dichloroaniline)
3236-63-3	2,2'-methylenebis(4-methylphenol)	2,2'-methylenebis(4-methylphenol)
2467-03-0	2,4'-dihydroxydiphenylmethane	2-(4-hydroxybenzyl)phenol
457-68-1	4,4'-difluorodiphenylmethane	bis(4-fluorophenyl)methane
101-68-8	4,4'-diphenylmethane diisocyanate, (4,4'-Methylenebis(phenyl Isocyanate))	bis(4-isocyanatophenyl)methane
139-25-3	4,4'-diisocyanato-3,3'-dimethyldiphenylmethane	bis(4-isocyanato-3-methylphenyl)methane
19471-12-6	3,3'-diaminodiphenylmethane	3,3'-methylenedianiline
2467-02-9	2,2'-dihydroxydiphenylmethane	2,2'-methylenediphenol
1844-01-5	4,4'-dihydroxytetraphenylmethane	4,4'-(diphenylmethylene)diphenol
174462-43-2	2,3,4,4'-tetrahydroxydiphenylmethane	4-(4-hydroxybenzyl)benzene-1,2,3-triol

Table S2.
PDB IDs of ERα utilized for calculating of the volumes of each ligand-binding pocket. PDB IDs are listed in alphabetical order.

PDB ID	ligand name	pocket volume (Å^3)
1A52	estradiol	389.6
1ERE	17beta-estradiol	461.4
1GWQ	raloxifene	425.4
1QKU	estradiol	407.1
1X7E	WAY-244	510.8
1X7R	genistein	457.9
2P15	ortho-trifluoromethylphenylvinyl estradiol	518.0
2QA6	4-(6-hydroxy-1*H*-indazol-3-yl)benzene-1,3-diol	318.1
2QGT	(9beta,11alpha,13alpha,14beta,17alpha)-11-(methoxymethyl)estra-1(10),2,4-triene-3,17-diol	417.8
2QSE	4-OH-PhIP	545.0
2QXM	PhIP	529.1
3Q95	estriol	422.0
3UU7	bisphenol A	428.0
3UUA	bisphenol AF	623.0
3UUC	bisphenol C	316.8
4MGA	4-tert-octylphenol	153.3
4MGC	benzophenone-2	314.0
4MGD	HPTE	395.1
4PP6	resveratrol	340.4
4PPP	fluoro-resveratrol	388.5
4PPS	(1*S*,3alpha*R*,5*R*,7alpha*S*)-5-(4-hydroxyphenyl)-7alpha-methyloctahydro-1*H*-inden-1-ol	466.4
4TV1	propylparaben	317.0
4ZN7	diethylstilbestrol	422.6
5DI7	(1*S*,3alpha*R*,5*S*,7alpha*S*)-5-(4-hydroxy-2-methylphenyl)-7alpha-methyloctahydro-1*H*-inden-1-ol	485.8
5DID	(1*S*,3alpha*R*,5*S*,7alpha*S*)-5-(2,3-difluoro-4-hydroxyphenyl)-7alpha-methyloctahydro-1*H*-inden-1-ol	460.4
5DIE	(1*S*,3alpha*R*,5*S*,7alpha*S*)-7alpha-methyl-5-(2,3,5-trifluoro-4-hydroxyphenyl)octahydro-1*H*-inden-1-ol	468.5
5DIG	(1*S*,3alpha*R*,5*S*,7alpha*S*)-5-[4-hydroxy-2-(trifluoromethyl)phenyl]-7alpha-methyloctahydro-1*H*-inden-1-ol	497.6
5EGV	3-chloranyl-4-[4-(2-chloranyl-4-oxidanyl-phenyl)furan-3-yl]phenol	395.0
5EI1	2-(4-hydroxyphenyl)-3-iodanyl-imidazo[1,2-alpha]pyridin-6-ol	389.9
5EIT	2-(4-hydroxyphenyl)-3-(trifluoromethyl)imidazo[1,2-alpha]pyridin-6-ol	381.4
5JMM	biochanin A	617.0
5KR9	coumestrol	350.9
5KRM	(1*S*,7alpha*S*)-5-(2,5-difluoro-4-hydroxyphenyl)-7alpha-methyl-2,3,3alpha,4,7,7alpha-hexahydro-1*H*-inden-1-ol	475.8
5KRO	(8*R*,9*S*,13*S*,14*S*,17*S*)-13-methyl-17-(methyl(phenyl)amino)-7,8,9,11,12,13,14,15,16,17-decahydro-6*H*-cyclopenta[alpha]phenanthren-3-ol	378.5
5TLL	(*E*)-2-chloro-4'-hydroxy-4-((hydroxyiminio)methyl)-[1,1'-biphenyl]-3-olate	359.5
5TLU	(14beta,17alpha)-21-(4-aminophenyl)-19-norpregna-1(10),2,4-trien-20-yne-3,17-diol	416.8
5TMZ	(8*S*,9*S*,13*S*,14*S*,17*S*)-16-(3-methoxybenzyl)-13-methyl-7,8,9,11,12,13,14,15,16,17-decahydro-6*H*-cyclopenta[alpha]phenanthrene-3,17-diol	436.8
5TN1	(8*S*,9*S*,13*S*,14*S*,*E*)-17-((4-isopropylphenyl)imino)-13-methyl-7,8,9,11,12,13,14,15,16,17-decahydro-6*H*-cyclopenta[alpha]phenanthren-3-ol	425.9

5TN3	(8*S*,9*S*,13*S*,14*S*)-17-((4-isopropylphenyl)amino)-13-methyl-7,8,9,11,12,13,14,15,16,17-decahydro-6*H*-cyclopenta[alpha]phenanthren-3-ol	475.9
5TN4	(*S*)-5-(4-hydroxy-3,5-dimethylphenyl)-2,3-dihydro-1*H*-inden-1-ol	388.9
5TN5	(1*S*,3alpha*S*,5*S*,7alpha*S*)-5-(4-hydroxyphenyl)-7alpha-methyloctahydro-1*H*-inden-1-ol	419.0
5TN6	(1*S*,1'*S*,3alpha'*S*,7alpha'*S*)-7alpha'-methyl-1',2,2',3,3',3alpha',4',6',7',7alpha'-decahydro-1,5'-spirobi[indene]-1',5-diol	448.0
5TN7	(*E*)-3'-fluoro-4'-hydroxy-3-((hydroxyiminio)methyl)-[1,1'-biphenyl]-4-olate	387.1
5TN8	(*E*)-4'-hydroxy-3-((hydroxyiminio)methyl)-[1,1'-biphenyl]-4-olate	355.3
5U2B	(8*R*,9*S*,13*S*,14*S*,17*S*)-13-methyl-17-(phenylamino)-7,8,9,11,12,13,14,15,16,17-decahydro-6*H*-cyclopenta[alpha]phenanthren-3-ol	515.8

Table S3.
PDB IDs of ERβ agonist structures utilized for calculating of the volumes of each ligand binding pocket. PDB IDs are listed in alphabetical order.

PDB ID	ligand name	pocket volume (Å^3)
1QKN	raloxifene	382.3
1U3Q	4-(6-hydroxy-benzo[delta]isoxazol-3-yl)benzene-1,3-diol	344.9
1U3R	2-(5-hydroxy-naphthalen-1-yl)-1,3-benzooxazol-6-ol	364.3
1U3S	3-(6-hydroxy-naphthalen-2-yl)-benzo[delta]isooxazol-6-ol	407.9
1X76	5-hydroxy-2-(4-hydroxyphenyl)-1-benzofuran-7-carbonitrile	354.4
1X78	[5-hydroxy-2-(4-hydroxyphenyl)-1-benzofuran-7-yl]acetonitrile	349.4
1X7B	2-(3-fluoro-4-hydroxyphenyl)-7-vinyl-1,3-benzoxazol-5-ol	314.0
1X7J	genistein	375.9
1YY4	1-chloro-6-(4-hydroxyphenyl)-2-naphthol	408.5
1YYE	3-(3-fluoro-4-hydroxyphenyl)-7-hydroxy-1-naphthonitrile	402.6
1ZAF	3-bromo-6-hydroxy-2-(4-hydroxyphenyl)-1*H*-inden-1-one	503.8
2J7X	estradiol	423.0
2J7Y	(16alpha,17alpha)-estra-1,3,5(10)-triene-3,16,17-triol	372.6
2NV7	4-(4-hydroxyphenyl)-1-naphthaldehyde oxime	340.6
2YJD	4-(2-propan-2-yloxybenzimidazol-1-yl)phenol	468.1
2YLY	*n*-cyclopropyl-4-oxidanyl-*n*-[(2*R*)-2-oxidanyl-2-phenyl-propyl]benzenesulfonamide	226.3
3OLL	estradiol	361.9
3OLS	estradiol	404.9
3OMO	2-(trifluoroacetyl)-1,2,3,4-tetrahydroisoquinolin-6-ol	289.0
3OMP	2-(trifluoroacetyl)-1,2,3,4-tetrahydroisoquinolin-7-ol	321.3
3OMQ	2-[(trifluoromethyl)sulfonyl]-1,2,3,4-tetrahydroisoquinolin-6-ol	346.9
4J24	estradiol	324.9
4J26	estradiol	434.6
4ZI1	2-(4-hydroxyphenyl)-7-methyl-3-phenyl-1*H*-inden-5-ol	350.4
5TOA	estradiol	361.0

Table S4.

Statistical analysis of agonistic activity data of BPA derivatives on ER β.

	0.1μM vs. 0	1 μM vs. 0	10 μM vs. 0	maximum fold induction		
**E2**	**	**	**	**9.41**	±	0.98
**1**	**	**	**	2.39	±	0.21
**2**	**	***	***	**5.31**	±	0.11
**3**	***	*	***	2.01	±	0.04
**4**	***	***	***	3.53	±	0.06
**5**	*	***	**	3.29	±	0.29
**6**	*	*	*	1.59	±	0.17
**7**			*	1.45	±	0.08
**8**	***	**	***	**5.13**	±	0.29
**9**		***	***	2.27	±	0.06
**10**	**	*		1.12	±	0.13
**11**		**	***	**4.25**	±	0.21
**12**		*		1.02	±	0.13
**13**			*	1.67	±	0.18
**14**	*	*		2.47	±	1.23
**15**			**	2.19	±	0.09
**16**			*	1.44	±	0.25
**17**			**	1.46	±	0.08
**18**	*	***	***	3.27	±	0.04
**19**	*	**	***	**9.00**	±	0.10
**20**				0.99	±	0.08

Table S5.

Statistical analysis of antagonistic activity data of BPA derivatives on ER β. Transcriptional activity indicated that the ratio of the transcriptional activity at most effective inhibitory concentration of each chemicals to that of 10 nM E2, which means full agonistic activity induced by E2.

	0.1μM vs. 0	1 μM vs. 0	10 μM vs. 0	transcriptional activity (%)		
**4OHT**	***	***	***	**5.4**	±	1.4
**1**	***	***	***	**6.0**	±	0.7
**2**	*	**	**	**21.6**	±	0.5
**3**		*	**	**42.5**	±	0.5
**4**	**	***	***	**22.9**	±	5.3
**5**	**	***	***	**19.9**	±	1.8
**6**	**	***	***	**11.1**	±	4.1
**7**	**	**	***	**6.6**	±	1.2
**8**		**	**	**28.9**	±	1.8
**9**			***	**17.0**	±	2.5
**10**	*	***	***	**5.4**	±	3.2
**11**	*	*	**	**22.4**	±	0.6
**12**		**	***	**7.7**	±	2.3
**13**				86.7	±	10.9
**14**	**		*	76.9	±	9.6
**15**		**	***	23.9	±	7.3
**16**	*	*	***	**11.8**	±	3.4
**17**	*	*		99.1	±	1.2
**18**	*	**	**	48.2	±	2.9
**19**		*	*	70.8	±	9.6
**20**		**	***	6.4	±	1.4

Table S6.

PDB IDs of ERβ LBDs utilized for SiteFinder calculations to analyze ligand-binding sites. PDB IDs are listed in alphabetical order.

PDB ID	rank of 1st site	rank of 2nd site	ligand	active or inactive	position of H12*
1HJ1	1	7	ICI164,384	inactive	free
1L2J	1	none	(*R*,*R*)-5,11-cis-diethyl-5,6,11,12-tetrahydrochrysene-2,8-diol	inactive	CBS
1NDE	1	none	4-(2-{[4-{[3-(4-chlorophenyl)propyl]sulfanyl}-6-(1-piperazinyl)-1,3,5-triazin-2-yl]amino}ethyl)phenol	inactive	CBS
1QKM	1	9	genistein	inactive	free
1QKN	1	10	raloxifene	inactive	free
1U3Q	1	none	4-(6-hydroxy-benzo[d]isoxazol-3-yl)benzene-1,3-diol	active	active position
1U3R	1	none	2-(5-hydroxy-naphthalen-1-yl)-1,3-benzooxazol-6-ol	active	active position
1U3S	1	none	3-(6-hydroxy-naphthalen-2-yl)-benzo[d]isooxazol-6-ol	active	active position
1U9E	1	3	2-(4-hydroxy-phenyl)benzofuran-5-ol	active	active position
1X76	1	3	5-hydroxy-2-(4-hydroxyphenyl)-1-benzofuran-7-carbonitrile	active	active position
1X78	1	none	[5-hydroxy-2-(4-hydroxyphenyl)-1-benzofuran-7-yl]acetonitrile	active	active position
1X7B	1	none	2-(3-fluoro-4-hydroxyphenyl)-7-vinyl-1,3-benzoxazol-5-ol	active	active position
1X7J	5	none	genistein	active	active position
1YY4	1	none	1-chloro-6-(4-hydroxyphenyl)-2-naphthol	active	active position
1YYE	1	17	3-(3-fluoro-4-hydroxyphenyl)-7-hydroxy-1-naphthonitrile	active	active position
1ZAF	1	3	3-bromo-6-hydroxy-2-(4-hydroxyphenyl)-1*H*-inden-1-one	active	active position
2FSZ	5	11	4-hydroxytamoxifen	inactive	free
2GIU	1	none	(9a*S*)-4-bromo-9a-butyl-7-hydroxy-1,2,9,9a-tetrahydro-3*H*-fluoren-3-one	inactive	CBS
2I0G	1	none	(3a*S*,4*R*,9b*R*)-4-(4-hydroxyphenyl)-1,2,3,3a,4,9b-hexahydrocyclopenta[c]chromen-8-ol	inactive	CBS
2I0J	1	9	(3a*S*,4*R*,9b*R*)-4-(4-hydroxyphenyl)-1,2,3,3a,4,9b-hexahydrocyclopenta[c]chromen-8-ol	inactive	free
2J7X	1	none	estradiol	active	active position
2J7Y	1	none	(16alpha,17alpha)-estra-1,3,5(10)-triene-3,16,17-triol	active	active position
2JJ3	1	none	(3a*S*,4*R*,9b*R*)-4-(4-hydroxyphenyl)-6-(methoxymethyl)-1,2,3,3a,4,9b-hexahydrocyclopenta[c]chromen-8-ol	inactive	CBS
2NV7	1	13	4-(4-hydroxyphenyl)-1-naphthaldehyde oxime	active	active position
2POG	1	8	(3a*S*,4*R*,9b*R*)-4-(4-hydroxyphenyl)-1,2,3,3a,4,9b-hexahydrocyclopenta[c]chromen-9-ol	inactive	free
2QTU	1	none	(3a*S*,4*R*,9b*R*)-2,2-difluoro-4-(4-hydroxyphenyl)-6-(methoxymethyl)-1,2,3,3a,4,9b-hexahydrocyclopenta[c]chromen-8-ol	inactive	CBS
2YJD	1	3	4-(2-propan-2-yloxybenzimidazol-1-yl)phenol	active	active position
2YLY	2	5	*N*-cyclopropyl-4-oxidanyl-*N*-[(2*R*)-2-oxidanyl-2-phenyl-propyl]benzenesulfonamide	active	active position
2Z4B	1	none	(3a*S*,4*R*,9b*R*)-2,2-difluoro-4-(4-hydroxyphenyl)-1,2,3,3a,4,9b-hexahydrocyclopenta[c]chromen-8-ol	inactive	CBS
3OLL	1	8	estradiol	active	active position

3OLS	1	none	estradiol	active	active position
3OMO	1	none	2-(trifluoroacetyl)-1,2,3,4-tetrahydroisoquinolin-6-ol	active	active position
3OMP	1	10	2-(trifluoroacetyl)-1,2,3,4-tetrahydroisoquinolin-7-ol	active	active position
3OMQ	1	none	2-[(trifluoromethyl)sulfonyl]-1,2,3,4-tetrahydroisoquinolin-6-ol	active	active position
4J24	1	10	estradiol	active	active position
4J26	1	9	estradiol	active	active position
4ZI1	1	none	2-(4-hydroxyphenyl)-7-methyl-3-phenyl-1*H*-inden-5-ol	active	active position
5TOA	1	7	estradiol	active	active position

* “CBS” means that H12 is located in an inactivated position on the ERβ coactivator-binding site (CBS); 'free' means helix 12 is not visualized or is far outside of the LBD.

Table S7.
Compounds names, CAS RN, and ligand IDs of CDS-Core or Chemical IDs from the Protein Data Bank (PDB); 3D coordinates were utilized for docking simulation experiments. Chemical IDs from PDB are designated by three letters.

	**common names**	**CAS RN®**	**Ligand ID**
**1**	bisphenol C	14868-03-2	0D1 (PDB)
**2**	4,4'-(1,3-dimethylbutylidene)bisphenol	6807-17-6	ZUHRAX
**3**	2,2-bis(p-hydroxyphenyl)-1,1,1- trichloroethane (HPTE)	2971-36-0	-
**4**	bisphenol AF	1478-61-1	TIBVOQ
**5**	bisphenol Z	843-55-0	-
**6**	4,4'-(2-ethylhexylidene)bisphenol	74462-02-5	-
**7**	4,4'-(2-hydroxybenzylidene)-bis(2,3,6-trimethylphenol)	184355-68-8	-
**8**	bisphenol B	77-40-7	-
**9**	1,1-bis(4-hydroxy-3-methylphenyl)cyclohexane	2362-14-3	SIJHOJ
**10**	bisphenol M	13595-25-0	-
**11**	bisphenol AP	1571-75-1	-
**12**	α, α, α'-tris(4-hydroxyphenyl)-1-ethyl-4-isopropylbenzene	110726-28-8	-
**13**	2,2-bis(3-amino-4-hydroxyphenyl)hexafluoropropane	83558-87-6	-
**14**	9,9-Bis(4-hydroxyphenyl)fluorene	3236-71-3	ABUCOP
**15**	9,9-bis(4-hydroxy-3-methylphenyl)fluorene	15499-84-0	XOGJEI
**16**	bisphenol P	2167-51-3	-
**17**	2,2-bis[4-(4-aminophenoxy)phenyl]hexafluoropropane	69563-88-8	HOYZOL
**18**	2,2-bis(4-hydroxy-3-methylphenyl)propane	79-97-0	REGKOF
**19**	bisphenol A	80-05-7	2OH (PDB)
**20**	α,α'-bis(4-hydroxy-3,5-dimethylphenyl)-1,4-diisopropylbenzene	36395-57-0	ACAYIN
